# Undiagnosed Sjögren's Syndrome Presenting as Mesenteric Panniculitis

**DOI:** 10.1155/2016/7207638

**Published:** 2016-06-05

**Authors:** Rebecca L. Burns, Sharukh J. Bhavnagri

**Affiliations:** ^1^Division of Rheumatology, University of Michigan Health System, Suite 7C27, North Ingalls Building, 300 North Ingalls Street, SPC 5422, Ann Arbor, MI 48109-5422, USA; ^2^Department of Diagnostic Radiology and Molecular Imaging, Oakland University William Beaumont School of Medicine, 3601 West 13 Mile Road, Royal Oak, MI 48073, USA

## Abstract

Mesenteric panniculitis is a rare inflammatory and fibrotic process that affects the small intestine mesentery. It may occur following abdominal surgery or in association with a variety of conditions, including malignancy, infection, and certain autoimmune and inflammatory conditions. Herein, an unusual case of mesenteric panniculitis in a patient with primary Sjögren's syndrome will be presented. The patient presented with abdominal pain, weight loss, sicca symptoms, fatigue, and arthralgia. An abdominal CT revealed mesenteric fat stranding and prominent lymph nodes of the small intestine mesentery. She was found on laboratory workup to have positive antinuclear and anti-SSa antibodies. Minor salivary gland lip biopsy revealed focal lymphocytic sialadenitis. The patient's symptoms and CT findings improved with corticosteroids. This case suggests that Sjögren's syndrome should be considered as an underlying disease process in the evaluation of patients with mesenteric panniculitis.

## 1. Introduction

Mesenteric panniculitis is an acute and chronic inflammatory and fibrotic process that affects the small intestine mesentery [1, 2]. It may be associated with a variety of conditions, including malignancy, prior abdominal surgery, infection, and certain autoimmune and inflammatory conditions [3]. Presenting symptoms include abdominal pain, bloating, weight loss, and intestinal obstruction [4]. Evaluation by CT may reveal soft tissue masses, prominent lymph nodes, and fibrosis or inflammation of the small bowel mesentery. Primary Sjögren's syndrome (pSS) is a chronic autoimmune disorder characterized by lymphocytic infiltration of salivary and lacrimal glands, resulting in severe sicca symptoms. pSS patients may also present with extraglandular features. A rare case of mesenteric panniculitis in a patient with pSS will be presented here.

## 2. Case Presentation

**Figure 1:**
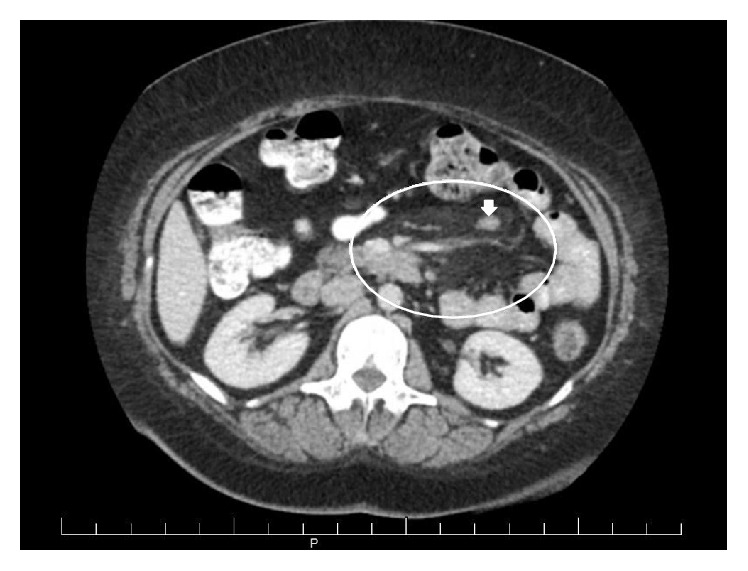
Axial CT image demonstrating mesenteric fat stranding (oval) with enlarged mesenteric lymph nodes (arrow) indicative of mesenteric panniculitis.

A 44-year-old female presented to rheumatology clinic with complaints of progressively worsening epigastric abdominal pain and distention over 7 months. She had previously been diagnosed with mesenteric panniculitis and was referred for further management. The abdominal pain began following a laparoscopic hysterectomy. At the time of rheumatologic consultation, she had lost 40 lbs. She complained of severe constipation and ability to have bowel movements only with heavy use of laxatives. She had a small amount of bright red blood in her stool intermittently, which had been attributed to hemorrhoids. Review of systems was negative for fevers, rashes, photosensitivity, oral or nasal ulcers, hair loss, or pleurisy. She admitted to having severe dryness of her eyes and mouth, diffuse arthralgia, fatigue, and chills. She had no history of renal failure, thrombosis, or miscarriage. EGD and colonoscopy performed previously were significant only for mild gastritis. A CT scan performed 5 months after the pain had begun revealed mesenteric fat stranding and prominent mesenteric lymph nodes concerning for mesenteric panniculitis (Figure 1).

Past medical history was significant for knee osteoarthritis, gastroesophageal reflux disease, and peptic ulcer with* H. pylori* infection. Prior surgeries preceding the laparoscopic hysterectomy included cholecystectomy, cesarean section, and partial hysterectomy. Family history was negative for rheumatic disease. Home medications included acetaminophen, lansoprazole, and laxative agents.

On physical exam, she was afebrile with normal vital signs. Mucous membranes were noted to be dry. Her abdomen was soft and nondistended, with moderate tenderness and guarding upon palpation over her epigastric region. Cardiac, pulmonary, neurologic, and joint exams were entirely unremarkable.

Laboratory workup revealed normal CBC, renal panel, and liver function panel. ESR was elevated at 54 mm. She had unremarkable urinalysis and urine protein-to-creatinine ratio. Antinuclear antibody (ANA) by immunofluorescence was positive with a titer of 1 : 640 in a speckled pattern. Anti-SSa antibody was positive. Anti-double stranded DNA (dsDNA), SSb, chromatin, Sm, RNP, scl-70, and centromere antibodies were all negative. Serum complements were normal, and serum protein electrophoresis revealed a polyclonal increase in gamma globulins, with no monoclonal protein present. Serum IgG4 level was 33.5 (normal range 8–150 mg/dL). A minor salivary gland biopsy revealed focal lymphocytic sialadenitis with a focus score of 1. A diagnosis of primary Sjögren's syndrome was made based on her presentation and laboratory findings.

**Figure 2:**
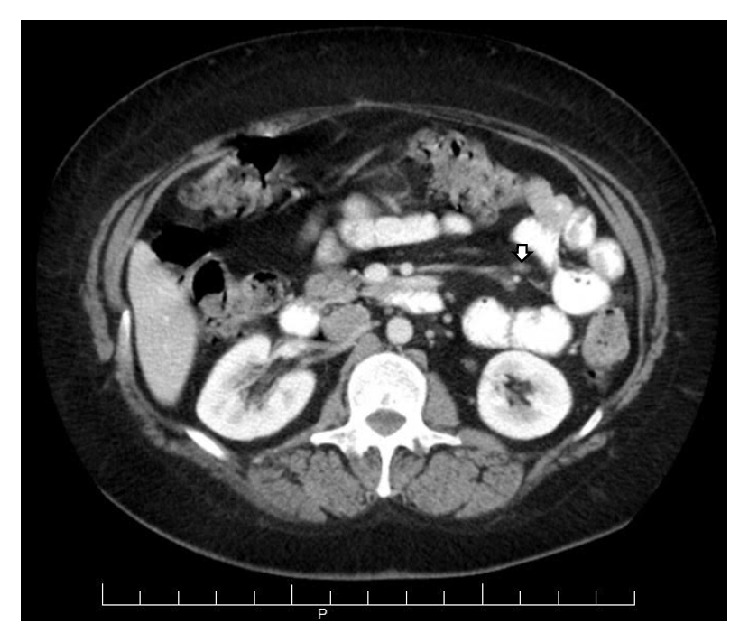
Axial CT image after therapeutic intervention reveals response to therapy with a decrease in the size of the mesenteric lymph node (arrow) and resolution of mesenteric fat stranding.

The patient was started on oral prednisone 60 mg daily. She had near-complete resolution of her abdominal symptoms and joint pain within weeks, and the steroid was tapered over 3 months. She was transitioned to colchicine. Hydroxychloroquine was initiated for pSS-related arthralgia and fatigue. Repeat CT scan showed improvement of the previously noted changes of mesenteric panniculitis, with decrease in size of the enlarged mesenteric lymph nodes and mesenteric fat stranding (Figure 2). She remains stable on colchicine and hydroxychloroquine.

## 3. Discussion

Mesenteric panniculitis is a rare inflammatory and fibrotic process that affects the small intestine mesentery [1, 2]. It may be associated with a variety of conditions, including malignancy, liver cirrhosis, infection, and certain autoimmune and inflammatory conditions [3]. It may also be associated with a prior history of abdominal surgery [4]. A variety of systemic and gastrointestinal symptoms may arise from the disease process, including abdominal pain, bloating, weight loss, nausea and vomiting, diarrhea, constipation, fever, and intestinal obstruction. A palpable abdominal mass or chylous ascites may also be present. The disorder usually occurs in middle or late adulthood with a slight male predominance [5]; however, one large study of 7620 abdominal CT scans showed a female predominance [3].

Various terms have been used in the description of mesenteric panniculitis, including retractile mesenteritis, mesenteric lipodystrophy, and sclerosing mesenteritis [5–8]. The different terms are likely more appropriate to describe different points in the progression of the disease; it begins as adipocyte necrosis (mesenteric lipodystrophy), followed by a chronic inflammatory state (mesenteric panniculitis), and finally progressing to fibrosis (sclerosing or retractile mesenteritis).

An abdominal CT is often used for diagnosis. CT findings may include a single or multiple soft tissue masses in the root of the mesentery, often with calcification [3, 4]. Rarely, infiltration of the pancreas or porta hepatis may occur [9]. There may be increased density of the mesenteric fat without a discrete mass, suggesting fibrosis or inflammation, as well as prominence of retroperitoneal and mesenteric lymph nodes. A hypodense fatty halo may be found surrounding vessels and nodules [3]. If there is significant involvement of the mesenteric vessels, bowel ischemia may result [9].

When biopsy is obtained, histopathologic features may include fibrosis, inflammation, and fat necrosis [4]. Lipid-laden foamy macrophages may be found infiltrating the mesenteric fat.

Mesenteric panniculitis may be associated with many autoimmune conditions, including primary sclerosing cholangitis, systemic lupus erythematosus (SLE), retroperitoneal fibrosis, and Riedel thyroiditis [9, 10]. Additionally, it has been suggested that the disease process may be associated with sclerosing pancreatitis and IgG4-related disease [4, 11].

The case presented here is an unusual one. Review of the literature revealed only 3 other case reports of mesenteric panniculitis associated with SS [12–14]. In addition, 2 patients with sclerosing mesenteritis from the study by Akram et al. had SS [4]. In the case presented by Batten and Ng, a 64-year-old male diagnosed with SS based on sicca symptoms and salivary gland biopsy underwent CT evaluation for abdominal pain and night sweats [13]. CT revealed edema of the mesenteric fat and lymph nodes in the jejunal mesentery. The patient improved symptomatically with a steroid taper; however, there was minimal improvement in the CT findings of mesenteric panniculitis. Sugihara et al. presented a case of a female with SS who developed abdominal pain, fever, and skin rash [12]. She was found to have systemic panniculitis involving the mesenteric adipose tissue as well as the skin. She also improved with corticosteroids alone. Osato et al. described a case of mesenteric panniculitis in a Human T-cell Lymphotropic Virus Type-I (HTLV-1) carrier with renal failure, pancytopenia, subcutaneous panniculitis, and uveitis [14]. The patient had a sicca syndrome thought to be HTLV-I associated SS. She had positive ANA with negative anti-SSa and anti-SSb antibodies. She underwent laparotomy for acute abdominal pain and was found to have mesenteric panniculitis with appendicitis. She later died from a mechanical ileus which developed at the surgical site.

It is important to exclude underlying malignancy in patients found to have mesenteric panniculitis. In the study by Daskalogiannaki et al., 69.4% of patients with mesenteric panniculitis were found to have a coexistent malignancy [3]. These included non-Hodgkin's lymphoma, breast and lung carcinomas, and various GI and gynecologic malignancies. Several patients had coexisting benign diseases, including SLE. Our patient was referred to general surgery for consideration of mesenteric biopsy to exclude lymphoproliferative process; however, she experienced such a dramatic improvement in symptoms and CT findings that a tissue diagnosis was thought to be unnecessary. She has undergone other age-appropriate cancer screenings without evidence for malignancy. She has had no other signs of symptoms to warrant further testing.

There is no standard treatment protocol for mesenteric panniculitis. Various treatment regimens have been proposed in small case series, including corticosteroids, tamoxifen, colchicine, azathioprine, thalidomide, and cyclophosphamide [4, 15–18]. Corticosteroids and other immunosuppressive agents appear to be the most promising due to their anti-inflammatory effects. While colchicine does not have a direct indication for the treatment of pSS, its efficacy in the management of mesenteric panniculitis, when combined with corticosteroids, has been demonstrated by Généreau et al. [15]. Fortunately, our patient improved with corticosteroids and was transitioned successfully to colchicine.

This case demonstrates the importance of considering Sjögren's syndrome as an underlying disease process in the evaluation of patients with mesenteric panniculitis.
